# Metabolic Changes of Hepatocytes in NAFLD

**DOI:** 10.3389/fphys.2021.710420

**Published:** 2021-08-30

**Authors:** Qianrang Lu, Xinyao Tian, Hao Wu, Jiacheng Huang, Mengxia Li, Zhibin Mei, Lin Zhou, Haiyang Xie, Shusen Zheng

**Affiliations:** ^1^Division of Hepatobiliary and Pancreatic Surgery, Department of Surgery, The First Affiliated Hospital, Zhejiang University School of Medicine, Hangzhou, China; ^2^NHC Key Laboratory of Combined Multi-organ Transplantation, Hangzhou, China; ^3^Department of Hepatobiliary and Pancreatic Surgery & Liver Transplantation, Shulan (Hangzhou) Hospital, Hangzhou, China

**Keywords:** WARBURG effect, NAFLD, Mitochondrial dysfunction, Insulin resistance, Metabolism

## Abstract

Nonalcoholic fatty liver disease (NAFLD) is often accompanied by systemic metabolic disorders such as hyperglycemia, insulin resistance, and obesity. The relationship between NAFLD and systemic metabolic disorders has been well reviewed before, however, the metabolic changes that occur in hepatocyte itself have not been discussed. In NAFLD, many metabolic pathways have undergone significant changes in hepatocyte, such as enhanced glycolysis, gluconeogenesis, lactate production, tricarboxylic acid (TCA) cycle, and decreased ketone body production, mitochondrial respiration, and adenosine triphosphate (ATP) synthesis, which play a role in compensating or exacerbating disease progression, and there is close and complex interaction existed between these metabolic pathways. Among them, some metabolic pathways can be the potential therapeutic targets for NAFLD. A detailed summary of the metabolic characteristics of hepatocytes in the context of NAFLD helps us better understand the pathogenesis and outcomes of the disease.

## Introduction

The liver is the hub of the metabolism of glucose, fat, protein, vitamin, hormone, etc., and play a major part in metabolic homeostasis. All kinds of metabolic processes such as glycolysis, gluconeogenesis, lipogenesis, glycogen production can be conducted in liver. Therefore, nonalcoholic fatty liver disease (NAFLD) is often accompanied by systemic metabolic disorders (Byrne and Targher, 2015; Tilg et al., 2017; Khan et al., 2019). In view of the importance and complexity of liver metabolism, it is meaningful to study the metabolic characteristics of the liver under healthy as well as pathological conditions.

Nonalcoholic fatty liver disease, a continuum of liver abnormalities including nonalcoholic fatty liver (NAFL) and nonalcoholic steatohepatitis (NASH), and can progress to liver cirrhosis and hepatocellular carcinoma (Friedman et al., 2018). NAFL, also named hepatic steatosis, is the benign stage of NAFLD which is characterized by excessive lipid stored within the cytosol of hepatocytes. Patients in the state of NAFL take a lower risk of deleterious outcomes, while NASH can progress to cirrhosis, liver failure and even hepatic carcinoma (Singh et al., 2015). The presence of NASH means the existence of liver inflammation and injury, accompanied with elevated aminotransferases. Biopsy is the main method of diagnosis which is characterized by various degrees of fat accumulation, hepatocellular ballooning, lobular inflammation and fibrosis (Brunt et al., 2020).

Nowadays NAFLD accounts for the major part of liver disease worldwide. With the decreased incidence of hepatitis and increased obesity (Fan et al., 2017; Wu et al., 2020), the primary cause of end-stage liver disease will be replaced by NAFLD in the next few decades (Younossi et al., 2018). Currently, the worldwide prevalence of NAFLD is nearly 24% (2015) (Younossi et al., 2016). Therefore there is an increasing interest in studying NAFLD. To understand the pathogenesis of NAFLD, a useful conceptual framework is that the excess energy metabolic substrates, fatty acids (FAs), and carbohydrates, exceed the liver’s processing capacity, resulting in the exacerbated formation of reactive oxygen species (ROS), by-products and lipid peroxidation, which will induce hepatocellular injury, inflammation and death (Friedman et al., 2018). In this review, we will discuss the hepatic metabolism changes and their effect in NAFLD.

## Carbohydrate Metabolism

### Glycolysis

Glycolysis refers to the process that one molecule of glucose is metabolized through a ten-step process in the cytoplasm to finally generate two molecules of pyruvate, providing with two molecules of NADH and two adenosine triphosphate (ATP). The glycolysis rate is mainly controlled by several rate-limiting enzymes, hexokinase (HK), phosphofructokinase (PFKM), and pyruvate kinase (PK), which are controlled by allosteric and hormonal regulation. In liver, the major isoform of hexokinase is hexokinase IV, glucokinase, which is primarily regulated by insulin but not inhibited by its product. Therefore, it is of great importance in the modulation of postprandial blood glucose. Insulin is the main hormone that regulates blood glucose. It bound to the insulin receptor (INSR) of target cells on the plasma membrane, then act through IRS(insulin receptor substrate)/PI3K(phosphoinositide 3-kinase)/AKT(protein kinase B) axis to regulate glucose metabolism (Haeusler et al., 2018; Petersen and Shulman, 2018). Phosphatase and tensin homolog (PTEN) catalyzed the reverse reaction of P13K and antagonize insulin function, but its activity is inhibited by insulin (Hodakoski et al., 2014).

Changes in glycolysis are found in many diseases. The WARBURG effect, known widely in tumor cells, is characterized by enhanced aerobic glycolysis and lactate production (Vander Heiden et al., 2009; Koppenol et al., 2011). It used to be misread that the damaged respiration is accounted for increased glucose fermentation in cancers. Actually, the tumor cells exhibit the WARBURG effect and meanwhile retain normal or even enhanced mitochondrial respiration (Koppenol et al., 2011; Lu et al., 2015). Recently, the similar metabolic change has also been found in many benign diseases, such as pulmonary hypertension, idiopathic pulmonary fibrosis (IPF), tuberculosis, failing heart (Chen et al., 2018), liver cirrhosis (Trivedi et al., 2021), etc., which indicate that WARBURG effect may not be unique to malignant tumor.

In the hepatocytes of fatty liver, similar metabolic changes also exist. Compared with regular chow (RC)-fed mice, the mRNA levels of key glycolysis related enzymes (HK2, PFKm, and PKm) were increased in the liver of high-fat diet (HFD)-fed mice (Liu et al., 2018). In the PTEN-null mice, HK2, PKM2, and other glycolytic enzymes are overexpressed via P13K/AKT2/PPARγ axis, which partially account for the susceptibility to fatty liver and hepatocellular carcinoma of PTEN-null mice (Horie et al., 2004). Moreover adenoviral-mediated overexpression of HK2 and PKM2 promotes liver growth and liver steatosis (Panasyuk et al., 2012), which may bridge the enhanced glycolysis with carcinogenesis in fatty liver. Using PET/CT to monitor the phosphorylation rate of liver glucose, which represents the activity of hexokinase, proved that the rate of phosphorylation of liver hexose is higher in patients with steatohepatitis (Keramida et al., 2016). Seahorse analysis also showed that the rate of extracellular acidification in HFD-fed mice increased suggesting enhanced glycolysis (Liu et al., 2018). Since NAFLD always accomplished by insulin resistance, hyperinsulinemia may be one of the reasons for enhanced glycolysis.

Recent studies have found that knocking down NOD-like receptor (NLR)X1 NLRX1 can inhibit glycolysis and enhance fat oxidation to reduce liver steatosis (Kors et al., 2018). Liver specific knockdown of SIRT6 will lead to the deacetylation of histone H3 lysine 9 (H3K9) in the promoter of glycolytic genes and elevated the expression of these genes, which consequently cause fatty liver dependent on enhancing glycolysis (Kim et al., 2010). Knockdown of Geranylgeranyl diphosphate synthase (GGPPS) can induce strengthened glycolysis through the LKB1–AMPK pathway, and lead to an increase in the inflammatory factors secreted in mouse primary hepatocytes. Furthermore, using 2-dexoy-D-glucose to inhibit glycolysis dramatically alleviates liver inflammation, injury and fibrosis due to the deletion of GGPPS in HFD mice (Liu et al., 2018). However, the pathway that glycolysis contributes to liver injury is unclear and deserves further study.

The product of glycolysis, pyruvate, will be dehydrogenated by lactate dehydrogenase to lactate or decarboxylated to acetyl-coenzyme A by pyruvate dehydrogenase complex (PDC) in mitochondria which thereby connects the glycolysis with the tricarboxylic acid (TCA) cycle (Zhou et al., 2001). The activity of the complex is regulated by pyruvate dehydrogenase phosphatase (PDP) and pyruvate dehydrogenase kinase (PDK1-4) through reversible dephosphorylation and phosphorylation. Phosphorylation is the inactive state of PDC (Bowker-Kinley et al., 1998).

The activity of PDC in hepatocytes of fatty liver is still controversial. Bajotto et al. (2006) and Go et al. (2016) found that the activity of pyruvate dehydrogenase complex in the liver of OLETF mice and HFD C57BL/6J mice was significantly lower than that of control group, which will consequently reduce the TCA cycle flux of pyruvate. Moreover, the activity, abundance and mRNA level of PDK2 and PDK4 are consistently increased, and positively related to plasma free fatty acid (FFA) concentrations (Bajotto et al., 2006; Go et al., 2016). Consistently, in the NAFLD patients and obese mice, the serum pyruvate concentration and liver pyruvate content are significantly higher than those of the control group (Fletcher et al., 2019; Wang et al., 2020; Shannon et al., 2021), though there is no evidence that it is caused by inactivity of PDC. A current study by Shannon et al. (2021) revealed a contradictory result that in obese mice, the activity of PDC is enhanced accompanied by the reduced activity of PDK4 and enhanced activity of PDP2. The insulin sensitizer Metformin can normalize the activity of PDC and mitigated the flow of TCA derived from pyruvate (Shannon et al., 2021). However, given that the acetyl-CoA produced by pyruvate only accounts for 5% of the liver TCA cycle (Cappel et al., 2019), the effect of pyruvate oxidative decarboxylation by PDC may not directly participate the pathological process of fatty liver.

### Lactate Production

The alternative metabolic pathway of pyruvate is lactate production. Under physiological conditions, lactate is mainly used by the heart, skeletal muscle and brain as an energy source (Gladden, 2004). The hepatocyte can convert excessive plasma lactate into glucose then bring back to plasma (Brooks, 2018). Lactate dehydrogenase(LDH) is composed of two subunits, H and M, with five isoenzymes: LDH1, LDH2, LHD3, LDH4, and LDH5 (Adeva-Andany et al., 2014). LDH-M, also known as LDHA, is more inclined to convert pyruvate into lactate; while LDH-H, also known as LDHB, is more inclined to convert lactate to pyruvate to dispose lactate (Dawson et al., 1964).

The production of lactate in fatty liver is significantly higher than normal (Liu et al., 2018; Wang et al., 2020), which is another feature of WARBURG effect (de la Cruz-López et al., 2019). P300/CBP-associated factor (PCAF)-mediated high acetylation level of LDH-B is one of the main causes in the accumulation of lactate in NAFLD. The activity of acetylated LDH-B is reduced, which hinders the ability of hepatocyte to dispose of lactate and results in the lactate accumulation (Wang et al., 2020). The increased lactate can not only worsen the hepatic steatosis, but also increase the acetylation of histone H3K9 by reducing the activity of nuclear histone deacetylase (HDAC), and therefore increase the expression of genes involved in lipogenesis and FA uptake (Wang et al., 2020).

### Liver Gluconeogenesis

Gluconeogenesis refers to the conversion of non-glucose compounds (glycerol, glycogen-producing amino acids, lactic acid, etc.) into glucose (Hers and Hue, 1983). Hepatic gluconeogenesis occurs during prolonged fasting episodes after depletion of hepatic glycogen, and is primarily in response to the insulin and glucagon. After an overnight fasting, about the half of the total hepatic glucose production is attributed to gluconeogenesis (Rothman et al., 1991). In order to achieve gluconeogenesis, the key is to bypass the three irreversible reactions in glycolysis that the conversion of glucose to glucose-6-phosphate by HK, fructose-6-phosphate to fructose-1,6-diphosphate by PFK and phosphoenolpyruvate to pyruvate via PK (Petersen et al., 2017). Conversion of pyruvate to phosphoenolpyruvate is the first reverse reaction in gluconeogenesis catalyzed by pyruvate carboxylase (PC) and phosphoenolpyruvate carboxykinase (PEPCK). The production of pyruvate carboxylation catalyzed by PC, oxaloacetate, is the intermediates of TCA cycle, and such flux is also named anaplerosis. Then oxaloacetate is converted to phosphoenolpyruvate, which is called cataplerosis (Owen et al., 2002). Thus, a close link exists between gluconeogenesis and TCA cycle. The enzymes that catalyze the other two reverse reactions are fructose diphosphatase-1 and glucose-6-phosphatase.

In the individuals with high intrahepatic triglycerides (IHTG), the endogenous glucose is higher than that in normal people, among which derived from gluconeogenesis is increased by 25%, consistent with the flow of anaplerosis increased by 50% (Sunny et al., 2011). The same trend also appears on mice supplied with excessive lipid, either by HFD or by exogenously perfused. Moreover, the increasement of anaplerosis/cataplerosis is linked to enhanced oxidative metabolism and subsequently oxidative stress and inflammation (Satapati et al., 2016). Inhibition of anaplerotic/cataplerotic flux can attenuates gluconeogenesis and TCA cycle and consequently alleviates oxidative stress and hepatic steatosis (Go et al., 2016; Satapati et al., 2016; Yuan et al., 2016). The presumable mechanism could be that, first, the raise of allosteric activation of PK, acetyl-COA, due to enhanced FA oxidation may partially contribute to the enhanced gluconeogenesis. Second, as mentioned before, the increased content of pyruvate in the liver increases the substrate for gluconeogenesis. Third the increased TCA cycle flux that we will discuss later facilitates gluconeogenesis from TCA cycle cataplerosis.

## Lipid Metabolism

### *De novo* Lipogenesis

Hepatic *de novo* lipogenesis (DNL) includes FAs synthesized from acetyl-CoA and then esterified with 3-phosphoglycerol to generate triglycerides (TG). Acetyl-CoA carboxylase (ACC) is the rate-limiting enzyme of FA synthesis, which carboxylate the acetyl-CoA to produce malonyl-CoA. Acetyl-CoA and citric acid are main allosteric activators of ACC. The expression and activity of the enzymes involved in DNL also modulated by various transcription factors like sterol regulatory element-binding protein 1C (SREBP1C) and carbohydrate-responsive element-binding protein (ChREBP; Shimomura et al., 1999; Dentin et al., 2005; Wang et al., 2015). Under physiological conditions, the TG synthesized in liver will not be stored in it, and will be exported in the form of very low density lipoproteins (VLDL; Kawano and Cohen, 2013).

The major feature of NAFLD is the lipid accumulation in hepatocytes. Under fasting conditions, the proportion of TG stored in liver of NAFLD patients are 26.1 ± 6.7% from DNL, 59.0 ± 9.9% from plasma free FAs and 14.9 ± 7.0% from dietary FAs, which is similar to the composition of VLDL-TG (Donnelly et al., 2005), and secretion rate of VLDL-TG is linearly related to the content of IHTG when IHTG content was ≤10% but reached a plateau in subjects with high IHTG (Fabbrini et al., 2008). Since NAFLD is often accompanied by insulin resistance (Watt et al., 2019), the lipolysis of peripheral adipose tissue increased, leading to an elevated in plasma FFA (Fabbrini et al., 2008; Samuel and Shulman, 2016). However, when compared to the individuals with low IHTG, no significant difference has been found in VLDL-TG derived from adipose tissue or diet in patients with high IHTG. Only the VLDL-TG derived from DNL was significantly increased to the three times of low IHTG group. The percentage of newly-synthesized palmitate in VLDL-TG was two times higher in high IHTG group, indicating that it was the increased DNL that contributed to the accumulation of liver fat (Lambert et al., 2014). Fabbrini et al. (2008) also proved that the increase in VLDL-TG secretion was primarily attributed to the FAs derived from DNL and lipolysis of abdominal and hepatic lipid, other than lipolysis of subcutaneous fat.

Moreover, the expression of lipogenic regulatory factors and enzymes, such as SREBP1c, fatty acid synthase (FAS), liver X receptor α (LXRα), ChREBP, and ACC1, is higher in fatty liver (Kohjima et al., 2007; Higuchi et al., 2008). LXRs are classified as the nuclear hormone receptor superfamily, which can be activated by oxysterols due to elevated intracellular cholesterol concentration. LXR is widely involved in the regulation of many metabolic processes including cholesterol, glucose and lipid metabolism (Hiebl et al., 2018). LXRα can promote lipogenic gene transcription by activating SREBP1c (Repa et al., 2000). The expression level of LXR in NAFLD patients was four times that of the control group, and it was significantly related with the expression of SREBP-1c (Higuchi et al., 2008). Moreover The expression of SREBP1c can also be induced by tumor necrosis factor-alpha (TNF-α) (Endo et al., 2007) and the mRNA level of TNF-α is related to the severity of NASH (Crespo et al., 2001). ChREBP is also found to overexpress in hepatic steatosis patient and indicate the severity of hepatic steatosis but is negatively correlated with insulin resistance in NASH patients. Overexpression of ChREBP induce the hepatic steatosis but increase the insulin sensitivity (Benhamed et al., 2012). In addition, due to the systemic insulin resistance, the high levels of insulin will contribute to DNL (Mansouri et al., 2018).

### Fatty Acid Oxidation

Fatty acids oxidation (FAO) is conducted in mitochondria, endoplasmic reticulum (ER) and peroxisomes which can produce abundant acetyl-CoA. The first step is activation of FAs to fatty acyl-CoA by acyl-CoA synthase in the outer mitochondrial membrane or ER. Mitochondria are the main site of FAs β-oxidation. The long-chain FAs rely on the assistance of carnitine palmitoyltransferase-1 (CPT1) to shuttled across the membrane while medium - and short-chain FAs do not. CPT1 is the key enzyme for β-oxidation, and malonyl-CoA, the intermediates of DNL, is a potent allosteric inhibitor of CPT (Bonnefont et al., 2004). Some transcription factors such as peroxisome proliferator-activated receptor α (PPARα) and its coactivator peroxisome proliferator-activated receptor gamma coactivator 1 α (PGC1α), can enhance the expression of CPT1 and other genes related to FAO (Bougarne et al., 2018). In the context of diabetes or FA overload, a part of FAs will be disposed through ω-oxidation in the endoplasmic reticulum which deteriorate ROS production and lipid peroxidation (Reddy, 2001; Wanders et al., 2011).

Theoretically increased DNL should inhibit the FA oxidation for the intermediate malonyl-CoA. However, in the patient with NASH or obesity, mitochondrial β-oxidation is still enhanced to coordinate with the excess FFA load, which has been proved by 13C-octanoate breath test and positron emission tomography and is correlated with systemic insulin resistance (Miele et al., 2003; Iozzo et al., 2010). In 12-week and 32-week HFD mice, the respiratory exchange ratio (ERE) value of the mice decreased and the oxygen consumption rate increased, suggesting increased FAO (Liu et al., 2018; Pittala et al., 2019). As the upregulation of fatty acid translocase (CD36/FAT), the uptake of FFA from plasma to hepatocytes also increased (Bechmann et al., 2010). CD36/FAT is one of the FA transport proteins which facilitates the uptake and utilization of FFAs in hepatocytes (Silverstein and Febbraio, 2009). Its expression is associated with serum FFAs and apoptosis mediators (Bechmann et al., 2010). The increased FFAs in hepatocytes could enhance the FAO consequently (Mansouri et al., 2018).

In fatty liver mouse model and NAFLD patients, the expression of genes involved in hepatic mitochondrial beta-oxidation such as PPARα, PGC1α and CPT1a is elevated as expected (Kohjima et al., 2007; Liu et al., 2018; Pittala et al., 2019). However, some research has shown that compared high-trans-fat high-fructose diet (TFD)-8 weeks-induced simple steatosis mice with NASH mice by 24 weeks, the expression of genes related beta-oxidation was decreased in liver with onset of NASH (Patterson et al., 2016). That may indicate that the ability of FAO may alter in different stages of NAFLD progression.

In addition, ω-oxidation conducted in the ER and β-oxidation in peroxisomes are upregulated in NASH as compensation (Robertson et al., 2001; Kohjima et al., 2007). However, it may lead to an increasement of ROS (Wanders et al., 2011; Del Río and López-Huertas, 2016). In addition, the increasement of the by-products of fat metabolism such as Cer and DAGs indicated that fat metabolism is insufficient during NSAH (Patterson et al., 2016), and these by-products will hinder insulin signaling pathway and result in insulin resistance (Samuel et al., 2010).

### Ketogenesis

There are three major flux for acetyl-CoA pool produced from FAO and glycolysis that ketogenesis, TCA cycle and DNL in liver. Ketone bodies are mainly synthesized by hepatocyte using acetyl-CoA derived from FAO. However, they cannot be used in liver for lack of enzymes that can utilize ketone bodies, and have to be exported to extrahepatic tissues for terminal oxidation (Puchalska and Crawford, 2017). This physiological process provides an alternative energy source which is enhanced when glucose is unavailable (Robinson and Williamson, 1980). Primary regulators of ketogenesis include the rate of mitochondrial β-oxidation, CPT1 activity, TCA cycle flux and its intermediate concentrations, and the hormones such as glucagon and insulin. When content of acetyl-CoA was elevated due to increased β-oxidation or impaired glycolysis reduced the oxaloacetate derived from anaplerosis, ketogenesis will strengthen as consequence.

Under physiological conditions, in terms of the ratio of TCA cycle and ketogenesis, 86 ± 2% of acetyl-CoA is used for ketone body production, while in patients with NAFLD, only 77 ± 2% of acetyl-CoA with *P* = 0.003 enters ketogenesis after a 24-h fast, and the serum β-hydroxybutyric acid concentration was 30% lower than that of the control group (Fletcher et al., 2019), even though there was no significant difference in the production of ketone bodies after overnight fasting (Sunny et al., 2011). In addition, the serum β-hydroxybutyric acid concentration was negatively correlated with the fat content in the liver (Fletcher et al., 2019). Compared with HFD-fed mice, ketogenic insufficiency aggravates the hepatic inflammation and injury in HDF-fed 3-hydroxymethylglutaryl CoA synthase (HMGCS2) antisense oligonucleotide (ASO)-treated mice. In addition, ketogenic insufficiency will increase DNL since more the acetyl-CoA will be catalyzed by citrate synthase to citrate (Cotter et al., 2014).

## Mitochondrial Metabolism

### TCA Cycle

Another metabolic pathway of acetyl-CoA is TCA cycle. The TCA cycle is a series of closed loop reactions. One molecule of acetyl-CoA that go through TCA cycle will produce 3 molecules of NADH, 1 molecule of FADH2, 2 molecules of C02 and 1 molecule of guanosine triphosphate GTP but the carbon does not directly drive from acetyl-CoA. Then NADH and FADH2 will be further oxidized by mitochondrial respiratory chain (MRC) and provide abundant ATP. The TCA cycle serves as the center of cell catabolism because no matter FA, protein or glucose, the terminal decomposition products are all acetyl-CoA. The regulation of TCA cycle mainly depends on allosteric regulation of the substrates, ATP, NADH, etc., but hardly by hormones (Martínez-Reyes and Chandel, 2020).

Under the fasting condition for 12 and 24 h, the acetyl-CoA flow into the TCA cycle in the liver of NAFLD patients was 2 and 1.4 times of normal people, respectively. which is also positively correlated with the liver triglyceride content, the rate of gluconeogenesis, and the blood glucose concentration (Sunny et al., 2011; Fletcher et al., 2019), although there is mitochondrial damage in term of morphology (Sanyal et al., 2001). Combined with attenuated ketogenesis we discussed before, we can draw the conclusion that the excess acetyl-CoA in hepatocytes of NAFLD patients was prone to be catabolized through the TCA cycle rather than ketogenesis, which reduced the fat consumption. Given that activity of TCA cycle is seldom controlled by hormones, and expression of related enzymes is constitutive. Therefore, the increased fat oxidation should be the main reason for TCA enhancement. The enhanced gluconeogenesis accompanied by enrichment of oxaloacetate that is able to activate pyruvate carboxylase may also contribute to overactive TCA cycle. Moreover the mitochondrial TCA cycle flux parallels to the ROS production and consequently cause oxidative stress (Satapati et al., 2012), thereby participate in the progression from liver steatosis to steatohepatitis (Browning and Horton, 2004). By inhibiting anaplerotic/cataplerotic flow, mitochondrial TCA flow and oxidative metabolism was reduced, and thereby mitigating inflammation and oxidative stress (Satapati et al., 2016). The enhancement of the TCA cycle increases the intermediates, citrate. It not only allosterically activates acetyl-CoA carboxylase, but also provides acetyl-CoA for DNL through the citrate pyruvate cycle.

### Mitochondrial Respiratory Chain

Embedded in the inner mitochondrial membrane, four kinds of protein complexes that complex I, II, III, IV make up the MRC which can transfer proton and electrons from NADH and FADH2. Then the oxygen molecules eventually accept electrons and hydrogen ions to produce water. During the delivery of electrons, protons are transported from the mitochondrial matrix at CI, CIII, and CIV and create proton-motive force (PMF). Then PMF is used to drive ATP generation by CV, coupling the MRC with ATP synthesis (Rich and Maréchal, 2010). As a consequence of normal mitochondrial respiratory, mitochondria generate ROS in a physiological range. Most of the electrons delivered to the MRC are correctly accepted by oxygen and then form water with protons catalyzed by cytochrome c oxidase (CIV). However, a small part of the electrons directly combined with oxygen to form superoxide anion radical (O_2_^–^) and other ROS, which usually happens in complex I and complex III (Willems et al., 2015; Mansouri et al., 2018). ROS in physiological level can activate various signaling pathways involved in cell proliferation, inflammation and etc. However, excessive ROS will attack intracellular macromolecular compound such as proteins, nucleic acids, lipids and lead to cell death or induce apoptosis (Zorov et al., 2014).

The efficiency of MRC also alters in NAFLD. In patients with obesity or simple fatty liver, hepatic mitochondrial respiration increased compared with healthy people. However, in patients with steatohepatitis, 31–40% of patients have lower hepatic mitochondrial respiratory levels than obese patients. It indicates that in the early stage of fatty liver, the respiration rate of mitochondria increases as compensation. When it progresses to the stage of steatohepatitis, the respiration of mitochondria begins to decrease, and this adaptive change disappears (Koliaki et al., 2015), and the activity of various complexes in the MRC of NASH patients is decreased consistently (Pérez-Carreras et al., 2003). As attenuated mitochondrial respiration is inadequate to the increased TCA cycle, which means the excess electrons cannot be captured by oxygen appropriately, NASH is always accompanied with more ROS production, DNA damage, inflammation and liver injury (Serviddio et al., 2008; Koliaki et al., 2015).

Moreover, in NASH patients and ob/ob mice, the expression of the uncoupling protein-2 (UCP-2) elevated, which aggravated the mitochondrial uncoupling and proton leakage of liver mitochondria, and reduced the efficiency of liver ATP synthesis (Chavin et al., 1999; Serviddio et al., 2008), All this leads to impaired ATP synthesis which has been validated in obesity and NAFLD patients. by nuclear magnetic resonance spectroscopy (Nair et al., 2003). Interestingly, uncoupling of liver mitochondria seems to play a protective role in the progress of fatty liver working by accelerating energy expenditure. Using adenine nucleotide translocase (ANT) inhibitor, carboxyatractyloside, can alleviate fatty liver and insulin resistance in mice by enhancing uncoupling (Cho et al., 2017).

## Therapeutic Strategies Targeted on Hepatocyte Metabolism

The treatment for NAFLD targeted on hepatocyte metabolism has demonstrated positive efficacy in some clinical trials. PPARα is one of the PPAR isotypes that mainly expressed in the liver, which can regulate liver FA uptake and the expression of FAO-related enzymes. Fibrates, PPARAα agonist, is mainly used to treat hyperlipidemia (Dubois et al., 2017). However, no obvious effect has been achieved in several clinical trials for the treatment of NAFLD. Nevertheless, several dual PPAR agonists have shown promising efficacy in recent clinical trials on the treatment of NAFLD. Saroglitazar, a dual PPAR α/γ agonist, has achieved a promising effect in the treatment of NAFLD in a phase 2 study(NCT03061721) (Gawrieh et al., 2021). Elafibranor, a dual PPARα/δ agonist, demonstrated the improvement of liver function tests and NASH resolution without worsening of fibrosis in a phase II study based on a post-hoc analysis(NCT01694849) (Ratziu et al., 2016). However, a phase III trial about it was terminated for not meeting the predefined primary surrogate efficacy endpoint (NCT02704403). FGF21 (fibroblast growth factor 21) is one of the target genes of PPARα, which mainly plays a role in the liver, such as enhancing FAO and reducing FA production (Kharitonenkov et al., 2005). Accordingly, Pegbelfermin, a FGF21 analogue, significantly reduced the hepatic fat fraction in patients with NASH determined by MRI-PDFF (magnetic resonance imaging-proton density fat fraction) in a phase 2a trial (NCT02413372) (Sanyal et al., 2019).

ACC is a key enzyme for lipogenesis. Therefore, it is reasonable to speculate that ACC inhibitors are able to reduce fatty liver. In a Phase 2 trial, GS-0976 (firsocostat), an ACC inhibitor, can significantly reduce DNL and ameliorate hepatic steatosis and markers of liver injury (NCT02856555) (Lawitz et al., 2018; Loomba et al., 2018). When combined with cilofexor (Farnesoid X Receptor agonist), firsocostat displayed greater improvement in hepatic steatosis and fibrosis of patients with advanced fibrosis caused by NASH defined by histology and clinically relevant biomarkers (NCT03449446) (Loomba et al., 2021).

Lifestyle intervention and weight loss are also effective methods to treat NAFLD. Among them, the ketogenic diet that increases the hepatic ketone production is popular in weight loss and is also helpful for NAFLD (Thoma et al., 2012; Saeed et al., 2019). In a clinical trial, 10 overweight/obese participants, who were restricted in carbohydrate intake while the intake of fat and protein remained unchanged, demonstrated decreased IHTG content and improved insulin sensitivity, despite increased circulating FFA (Luukkonen et al., 2020).

There is no FDA-approved drug for NAFLD treatment so far. Therefore, it is of great interest to explore novel targets for NAFLD treatment. In animal models, changes in glycolysis, lactate production, and mitochondrial uncoupling can affect the progression of NAFLD, which can be the potential pharmacological targets for NAFLD.

## Conclusion

Although the metabolism of liver is generally regulated by insulin and glucagon, NALFD is often accompanied by systemic insulin resistance and the insulin resistance is selective (Brown and Goldstein, 2008), which makes the actual metabolic state hard to predict. In this review we found that the changes in DNL, glycolysis, and ketogenesis is in accord with insulin effect, whereas gluconeogenesis and FAO is less inhibited by insulin.

Therefore, it is meaningful to study the specific changes in metabolism and their role in the pathogenesis of NAFLD. In this review, we mainly discussed changes in lipid and glucose metabolism. Glycolysis is significantly enhanced in the hepatocytes of fatty liver, resulting in the increased content of pyruvate in plasma and liver. Enhanced glycolysis can cause liver inflammation, but the specific pathway is still unclear. PDC is the main enzyme that controls the flux of pyruvate into the TCA cycle. The alteration of its activity in fatty liver is still controversial. Pyruvate can also be converted to oxaloacetate through anaplerosis that provides intermediates for TCA, or to lactate, both of which are enhanced in NAFLD. Increased lactate will elevate the expression of lipogenic enzymes dependent on acetylation of H3K9, and aggravate NAFLD.

Excessive accumulation of fat in hepatocyte is the fundamental feature of NAFLD, accompanied by an increase in VLDL secretion, which is mainly derived from strengthened DNL. Meanwhile, the FAs oxidation is also enhanced in the mitochondria as well as the peroxisomes and ER. The oxidation that occurs in the latter can lead to more production of ROS that causes inflammation and liver damage.

**FIGURE 1:**
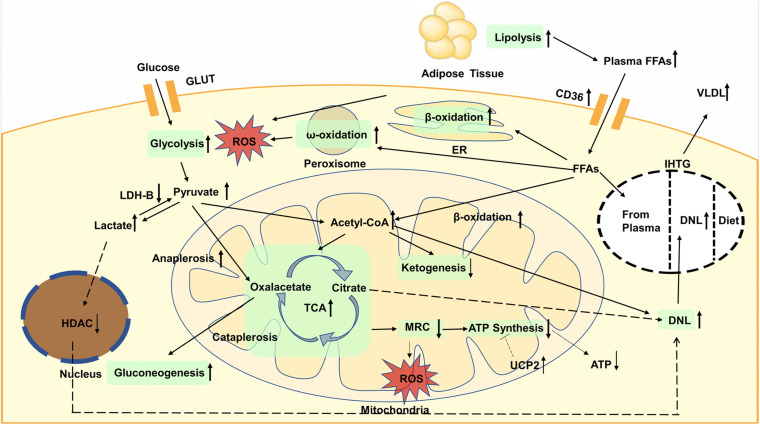
Glycolysis is enhanced, resulting in the increased content of pyruvate in plasma and liver. Then pyruvate is converted to oxaloacetate through anaplerosis or to lactate, both of which are enhanced in NAFLD. Increased lactate will elevate the expression of lipogenic enzymes dependent on decreased activity of nuclear HDAC. Gluconeogenesis is increased and contribute to TCA cycle. Lipolysis is increased, leading to elevated plasma FFAs. accumulation of TAGs is mainly caused by increased DNL. The oxidation of fatty acids is also enhanced, not only in the mitochondria, but also in the endoplasmic reticulum and peroxisomes. The oxidation that occurs in the latter can lead to more production of ROS that causes inflammation and liver damage. Increased glycolysis and fatty acid oxidation will elevate acetyl-CoA and enhance the TCA cycle whereas ketogenesis is reduced. The activity of MRC is reduced, resulting in more ROS generation. The expression of UCP2 increased in fatty liver, which impaired efficiency of ATP synthesis and decreased ATP content. GLUT, glucose transporter; FFAs, plasma free fatty acids; CD36, cluster of designation 36; ROS, reactive oxygen species; ER, endoplasmic reticulum; VLDL, very low density lipoproteins; IHTG, intrahepatic triglycerides; DNL, *de novo* lipogenesis; LDH, lactate dehydrogenase; HDAC, histone deacetylase; TCA, tricarboxylic acid; MRC, mitochondrial respiratory chain; ATP, adenosine triphosphate; UCP, uncoupling protein.

Increased glycolysis and FAO will elevate acetyl-CoA and enhance the TCA cycle, which will increase liver mitochondrial burden. However, the production of ketone bodies is reduced, indicating that the acetyl-CoA is more inclined to enter the flux of TCA cycle. Nevertheless, the activity of MRC is reduced, which cannot match the enhanced TCA cycle, resulting in more ROS generation. Moreover, the expression of UCP2 also increased in fatty liver, which impaired efficiency of ATP synthesis. The global metabolic changes are shown in Figure 1.

## Author Contributions

QL, XT, LZ, and SZ: conception. QL, XT, HW, JH, ML, and ZM: drafting manuscript. XT, HX, LZ, and SZ: revising manuscript. All authors contributed to the article and approved the submitted version.

## Conflict of Interest

The authors declare that the research was conducted in the absence of any commercial or financial relationships that could be construed as a potential conflict of interest.

## Publisher’s Note

All claims expressed in this article are solely those of the authors and do not necessarily represent those of their affiliated organizations, or those of the publisher, the editors and the reviewers. Any product that may be evaluated in this article, or claim that may be made by its manufacturer, is not guaranteed or endorsed by the publisher.
